# The first complete mitochondrial genome of *Phyrella fragilis* (Mitsukuri & Ohshima in Ohshima, 1912) (Dendrochirotida: Phyllophoridae)

**DOI:** 10.1080/23802359.2021.1976687

**Published:** 2021-09-17

**Authors:** Shengping Zhong, Longyan Zhao, Guoqiang Huang, Yonghong Liu, Lianghua Huang

**Affiliations:** a Institute of marine drugs, Guangxi University of Chinese Medicine, Nanning, China; b Guangxi Engineering Technology Research Center for Marine Aquaculture, Guangxi Institute of Oceanology Co., Ltd, Beihai, China

**Keywords:** Mitochondrial genome, *Phyrella fragilis*, Phyllophoridae

## Abstract

Echinoderms (Echinodermata) are morphologically diverse and ecologically important groups of marine invertebrate, many of which are key components of local benthic ecosystem. However, due to morphological plasticity and limited molecular phylogenetic studies, the taxonomic histories in echinoderms have not been completely resolved. The phylogenetic relationships of Phyllophoridae genera and species remain controversial and many species are incorrectly assigned within genus *Phyrella*. In this study, we report the first complete mitochondrial genome of *Phyrella* from *Phyrella fragilis*. The mitogenome has 15,910 base pairs (64.32% A + T content) and is made up of a total of 37 genes (13 protein-coding, 22 transfer RNAs and 2 ribosomal RNAs), plus a putative control region. This study will provide useful genetic data for future phylogenetic and taxonomic classification of Phyllophoridae.

Sea cucumbers (Holothuroidea) also known as holothuroids, belong to the phylum Echinodermata, which are species diverse groups of marine benthic invertebrates (Miller et al. 2017). Sea cucumbers contain more than 1763 living species (WoRMS 2021) with a global distribution in almost every marine environment (Kerr and Kim 2001), many of which are economically important fisheries resources for their valuable nutrition and healthy pharmacological compounds (Zhong et al. 2020). Due to their diversity and commercial importance, sea cucumbers have been subject of intense research resulting in new data on their phylogeny (Miller et al. 2017). The genus *Phyrella* which belong to family Phyllophoridae had been redefined recently, five species including *Phyrella fragilis* had been recognized in *Phyrella* (Michonneau and Paulay 2014). However, due to complex taxonomic characteristics and insufficient molecular data, the phylogenetic relationship within Phyllophoridae remains poorly explored, and the mitochondrial genomes of *Phyrella* had not been sequenced yet. Genetic data have proven to be useful for understanding phylogenetic relationship within Phyllophoridae (Yang et al. 2020). Here, we report the first complete mitochondrial genome of *Phyrella* holothuroids, which will afford useful molecular information for taxonomic and phylogenetic analyses in sea cucumbers.

Tissue samples of *P. fragilis* from one individuals were collected from HaiNan province, China (SanYa, 18.310365 N, 109.798831 E) by local diving fishermen and the whole body specimen (#JP0116) was deposited at Marine biological Museum, Guangxi Institute of Oceanology, Beihai, China (http://www.gxas.cn/kypt/kxpj/kpcg, Shengping Zhong, shpzhong@foxmail.com). The total genomic DNA was extracted from the muscle of the specimen using an SQ Tissue DNA Kit (OMEGA, Guangzhou, China) following the manufacturer’s protocol. DNA libraries (350 bp insert) were constructed with the TruSeq NanoTM kit (Illumina, San Diego, CA) and were sequenced (2 × 150 bp paired-end) using HiSeq platform at BGI Company, China. Mitogenome assembly was performed with MITObim (Hahn et al. 2013). The cytochrome oxidase subunit I (COI) gene of *P. fragilis* (GenBank accession number: JX544959) (Michonneau and Paulay 2014) was chosen as the initial reference sequence for MITObim assembly. Gene annotation was performed by MITOS (Bernt et al. 2013).

The complete mitogenome of *P. fragilis* (GenBank accession number: MZ305459) is 15,910 bp in length and it contains a conserved set of 13 protein-coding genes (PCGs), 2 ribosomal RNA genes, 22 transfer RNA genes, and a putative control region. A total of 37 genes were annotated and 589 nucleotides were identified as putative control region. The overall base composition of the mitogenome is estimated to be A 36.44%, T 27.89%, C 23.42% and G 12.26%, with a high A + T content of 64.32%, which is similar, but slight lower than *Phyllophorus liuwutiensis* (65.19%) (Yang et al. 2020) within family Phyllophoridae. The phylogenetic analysis inferred from the concatenated nucleotides sequences of 13 PCGs also suggests that *Phyrella* and *Phyllophorus* holothuroids have closely relationship in family Phyllophoridae (Figure 1), which is consistent with the phylogenetic analyses of Phyllophoridae holothuroids using DNA barcoding sequences (Michonneau and Paulay 2014). The complete mitochondrial genome sequence of *P. fragilis* was the first sequenced mitogenome in *Phyrella* holothuroids, which will be useful for better resolving the genera and subfamilies controversy in Phyllophoridae.

**Figure 1.**
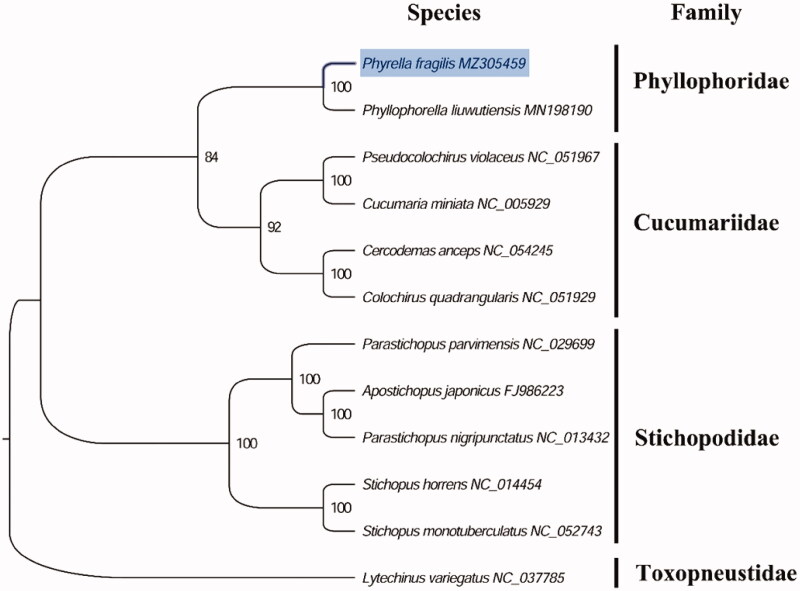
Phylogenetic tree of 12 species in echinoderms. The complete mitogenomes were downloaded from GenBank and the phylogenic tree based on the concatenated nucleotide sequences of 13 mitochondrial PCGs was constructed by maximum-likelihood method via PhyML online server (http://www.atgc-montpellier.fr/phyml/), using GTR substitution model with 100 bootstrap replicates. The bootstrap values are indicated at each branch nodes, echinoid (Lytechinus variegatus) was rooted to be outgroup species.

## Data Availability

The genome sequence data that support the findings of this study are openly available in GenBank of NCBI at (https://www.ncbi.nlm.nih.gov/) under the accession no. MZ305459. The associated BioProject, SRA, and Bio-Sample numbers are PRJNA733827, SRR14692230, and SAMN19460001, respectively.
